# Editorial: Emerging research: self-ascribed parapsychological abilities

**DOI:** 10.3389/fpsyg.2025.1596390

**Published:** 2025-04-10

**Authors:** Luca Simione, Camilla Pagani, Andrew Denovan, Neil Dagnall

**Affiliations:** ^1^Dipartimento di Scienze Umanistiche e Sociali Internazionali, Università degli Studi Internazionali (UNINT), Rome, Italy; ^2^Istituto di Scienze e Tecnologie della Cognizione, Consiglio Nazionale delle Ricerche (CNR), Rome, Italy; ^3^School of Psychology, Liverpool John Moores University, Liverpool, United Kingdom; ^4^School of Psychology, Manchester Metropolitan University, Manchester, United Kingdom

**Keywords:** parapsychological experience, parapsychological phenomena, self-ascribed paranormal ability, personality, altered state of consciousness

Since paranormal beliefs provide insights into cognitive biases, personality traits, and existential concerns, they have long been a subject of psychological research. Studies have demonstrated that such beliefs are associated with intuitive thinking, reduced analytical reasoning, and susceptibility to cognitive illusions (Lindeman and Aarnio, 2007). Additionally, researchers report correlations between paranormal beliefs and schizotypal personality traits, suggesting that these convictions stem, at least in part, from cognitive-perceptual tendencies that shape individual interpretations of reality (Dagnall et al., 2010; Williams and Irwin, 1991). Beyond cognitive factors, theorists link paranormal beliefs to emotional and existential dimensions, functioning as coping mechanisms for uncertainty and mortality-related anxieties (Dagnall et al., 2025; Lange and Houran, 1999).

Embedded within these broader belief systems is the notion of paranormal abilities (i.e., extraordinary capacities such as mind-to-mind communication, anomalous perception of distant events, and the power to influence physical matter). Historically, these capabilities have been the focus of parapsychology, which has examined their existence and potential mechanisms (Irwi and Watt, 2007). However, contemporary research has shifted focus from proving or disproving paranormal abilities to understanding their psychological underpinnings (Roxburgh et al., 2024). Scholars now explore the cognitive, affective, and cultural factors that shape the subjective experience of paranormal phenomena and consider their impact on sense of self and agency (Simmonds-Moore).

A key development in this respect is the study of self-ascribed paranormal abilities (i.e., claims by individuals who personally possess such extraordinary capabilities). Unlike paranormal beliefs, which people hold abstractly or vicariously, self-ascribed abilities are rooted in direct personal experience and are typically integrated within identity and worldview (Drinkwater et al., 2022). Research suggests that individuals who self-ascribe paranormal abilities exhibit heightened cognitive-perceptual tendencies, such as anomalous perception and absorption, which contribute to their conviction in experiences (Goulding, 2005; Krippner and Friedman, 2010). Moreover, social and cultural factors shape these self-attributions, with media representations and personal narratives reinforcing belief structures (Simmonds-Moore, 2016). Despite increasing methodological rigor in this field, further research is necessary to disentangle the cognitive and emotional processes underlying self-ascribed paranormal abilities. By adopting an interdisciplinary approach that integrates psychology, neuroscience, and contemplative studies, scholars can gain deeper insights into why these experiences emerge, how they shape belief systems, and what they reveal about the construction of human perception and consciousness.

Building on this evolving research field, this Research Topic deepens understanding of self-ascribed experiences by bringing together innovative contemporary contributions. Specifically, it provides a platform for examining how individuals perceive, interpret, and integrate parapsychological experiences into their psychological frameworks. By incorporating empirical studies, theoretical analyses, and interdisciplinary perspectives, this initiative reflects the increasing complexity of investigations into self-ascribed paranormal abilities. Accordingly, the collected works explore a range of dimensions, from cognitive and personality correlates to broader cultural and existential implications.

One major area of research concerns the cognitive and personality traits associated with self-ascribed paranormal abilities. In this respect, Dagnall et al. examined relationships between paranormal belief and conspiracy theory endorsement, with an emphasis on their impact on wellbeing. Findings revealed that paranormal beliefs were associated with both active and passive coping mechanisms, contributing to a greater sense of meaning in life. In contrast, conspiracy theory endorsement linked to social identity and avoidant coping strategies. This distinction highlights how paranormal beliefs, including self-ascribed parapsychological abilities, serve adaptive psychological functions by offering individuals frameworks for meaning-making and coping.

Simmonds-Moore et al. further expanded this observation by exploring psychometry, the claimed ability to gain information through touch, and its associations with synesthesia and Autonomous Sensory Meridian Response (ASMR). Findings indicated a potential overlap between psychometric experiences and these sensory phenomena, with individuals reporting heightened sensory sensitivity more likely to experience or interpret psychometric events. These associations suggest that psychometry stems from enhanced sensory processing which contributes to anomalous experiences.

Another area of study regards the exploration of self-ascribed parapsychological abilities through altered states of consciousness, which reveals important insights into the intersection of cognitive, neural, and phenomenological processes. A comparative analysis of naturalistic N,N-Dimethyltryptamine (DMT) experiences and NDEs by Michael et al. identified thematic parallels, particularly concerning themes of death and dying. Both experiences commonly involved encounters with sentient entities, transcendence of time and space, and ego dissolution. These findings suggest that both DMT-induced experiences and NDEs share profound phenomenological similarities, shedding light on how certain altered states can evoke intense, death-related imagery and transcendental perceptions.

In the same vein, Toriz et al. took a neuroanthropological approach to shamanic trance, examining the brain activity of a ritual specialist in Mexico. Using electroencephalography, they observed significant beta and gamma oscillations during the trance state, which may reflect neuroplastic phenomena that modulate the assimilation of sensory and cognitive references. This study emphasized the role of cultural practices in shaping non-ordinary states of consciousness and highlighted how cultural beliefs and rituals influence the human brain's inherent potential to enter these states.

Finally, the systematic review and meta-analysis by Chaudhary et al. aimed to investigate the brain activity linked to arousal or wake-promoting effects during Buddhist meditation using fMRI studies. The analysis highlighted the activation of brain regions associated with alertness, emotion regulation, and attention, particularly in areas like the prefrontal cortex and insula. The findings suggest that Buddhist meditation practices can promote a state of heightened awareness and alertness, potentially offering insights into how meditation affects mental states related to arousal and wakefulness.

The study of self-ascribed paranormal abilities, such as psychometry and other parapsychological experiences, reveals that these abilities link closely to cognitive and personality traits, including heightened perceptual sensitivity and susceptibility to anomalous experiences. Research shows that these beliefs often serve adaptive psychological functions, helping individuals cope with existential concerns. The connection between altered states of consciousness, like those induced by DMT or shamanic trance, and self-ascribed abilities suggests a shared phenomenology centered on death, transcendence, and ego dissolution. Future research could delve deeper into the neural mechanisms behind these phenomena and their role in mental health.
